# The Standardized Pediatric Expedited Encounters for ART Drugs Initiative (SPEEDI): description and evaluation of an innovative pediatric, adolescent, and young adult antiretroviral service delivery model in Tanzania

**DOI:** 10.1186/s12879-018-3331-2

**Published:** 2018-09-03

**Authors:** Jason M. Bacha, Lynda C. Aririguzo, Veronica Mng’ong’o, Beatrice Malingoti, Richard S. Wanless, Katherine Ngo, Liane R. Campbell, Gordon E. Schutze

**Affiliations:** 1 Pediatrics, Baylor College of Medicine Children’s Foundation – Tanzania, Centre of Excellence at Mbeya Zonal Referral Hospital, Box 2663, Mbeya, PO Tanzania; 2 Baylor International Pediatric AIDS Initiative (BIPAI) at Texas Children’s Hospital, Baylor College of Medicine, Houston, TX USA; 3 Department of Pediatrics, Baylor College of Medicine, Houston, TX USA

**Keywords:** Pediatric HIV/AIDS, ART delivery, Expedited encounters, Differentiated models of care, Lost to follow up, Mortality

## Abstract

**Background:**

As countries scale up antiretroviral therapy (ART) for children, innovative strategies to deliver quality services to children are needed. Differentiated ART delivery models have been successful in adults, but no such program has been described in children. We describe the Standardized Pediatric Expedited Encounters for ART Drugs Initiative (SPEEDI).

**Methods:**

Descriptive analysis of patients eligible for SPEEDI was done via retrospective review of children, adolescents, and young adults on ART at the Baylor Centre of Excellence (COE) in Mbeya, Tanzania between January 2013 and December 2015. Eligibility for SPEEDI visits included the following: stable children, adolescents, and young adults on ART for approximately 3 months or longer, no medical or social complications, good adherence to ART, and presence of reliable caregiver. During a SPEEDI visit, patients were fast tracked in triage to collect medications directly without physically seeing a clinician. SPEEDI patients came to clinic every two months, and alternated SPEEDI visits with standard visits. Baseline characteristics, mortality, and lost-to-follow up rates of SPEEDI patients were analyzed.

**Results:**

One thousand one hundred sixty-four patients utilized SPEEDI, totaling 3493 SPEEDI visits. SPEEDI reached 51.3% (1164/2269) of pediatric ART patients, accounting for 7.7% (3493/44489) of total patient encounters. SPEEDI patients were 52% (605/1164) female, median age of 11.7 years (range 1.2–25.5 yr), median time on ART of 21 months (range 4–130 months) and 83.5% (964/1155) categorized as no or mild HIV-associated immunodeficiency. SPEEDI patients had good outcomes (98.8%), low LTFU (0.1%) and low mortality rates (0.61 deaths per 100 patient-years).

**Conclusion:**

SPEEDI was an effective model for delivering ART to children, adolescents, and young adults in our setting, leading to good clinical outcomes, low mortality, and low LTFU. The SPEEDI program safely and effectively expedited and spaced out ART visits for children, adolescents, and young adults, and can serve as an adaptable ART delivery model for other resource limited settings.

## Background

The burden of HIV/AIDS in children remains high as demonstrated by the 150,000 new infections, 110,000 AIDS-related deaths, and low antiretroviral therapy (ART) coverage (only 49% of an estimated 1.8 million children living with HIV have accessed ART) reported worldwide in 2015 in children under the age of 15 years [1]. In Tanzania, the treatment gap is even wider with only 16% of an estimated 200,000 children living with HIV are on ART [2]. Despite significant progress in these areas since 2000, there are still major challenges in reaching the 90–90-90 targets for children living with HIV [3].

As countries scale up availability and provision of ART for children and adolescents, innovative strategies to effectively deliver quality services to substantially more clients without overwhelming already overburdened clinics are needed. Differentiated approaches to HIV care have been promoted as an effective way to put the patient at the centre of service delivery while reducing unnecessary burdens on the health systems [4, 5]. Examples of differentiated care models – such as community ART groups and spacing of clinic visits – have been effective in adults in reducing patient and health system burdens while improving clinical care and outcomes [6–12]. However, evidence and descriptions of differentiated care models in children and adolescents is lacking, especially in young children and in resource limited settings [13, 14]. As increasing numbers of children and adolescents access ART through 90–90-90 efforts, innovative models of care tailored to the unique characteristics and needs of children and adolescents need to be implemented and explored.

One such model of care aiming to optimize service delivery to children and adolescents living with HIV is the Standardized Pediatric Expedited Encounters for ART Drugs Initiative (SPEEDI). SPEEDI utilizes appointment spacing and fast-track ART drug refills, and was designed and piloted at the Baylor College of Medicine Children’s Foundation – Tanzania Centre of Excellence (COE) in Mbeya, Tanzania. We provide a descriptive analysis of the SPEEDI program and the lost-to-follow up (LTFU) and mortality outcomes for children, adolescents, and young adults on ART utilizing the SPEEDI program.

## Methods

### Ethical approval

Approval was obtained from the Mbeya Medical Research and Ethics Committee and the Medical Research Coordinating Committee of the National Institute for Medical Research (NIMR) in Tanzania, and the Institutional Review Board, Baylor College of Medicine in Houston, Texas, USA (H-32491). Waiver of patient informed consent was approved by all committees as this retrospective study analyzed only de-identified data.

### Study setting and description of SPEEDI model

The Baylor College of Medicine Children’s Foundation – Tanzania COE is located in Mbeya, Tanzania offering comprehensive pediatric HIV care. The Baylor-Mbeya COE opened in 2011, has treated more than 5000 HIV affected children and adolescents, and currently cares for over 2500 patients active in care. The Baylor-Mbeya COE is located at the Mbeya Zonal Referral Hospital (MZRH), the zonal hospital for the Southern Highlands Zone of Tanzania, with a catchment area of 3.2 million children [15]. Children, adolescents, and young adults receive HIV care at the COE while caregivers and adults receive HIV care at the neighboring adult HIV clinic on site at MZRH. Family-centered care is provided through alignment of appointment dates for both children and caregivers. The SPEEDI program was implemented in January 2013 in response to high patient volumes as a means to decongest the clinic while maintaining high quality services. Prior to SPEEDI implementation, all patients on ART were dispensed one month supply of medications, given one month follow up visits, and were seen by physicians at every ART visit.

**Fig. 1:**
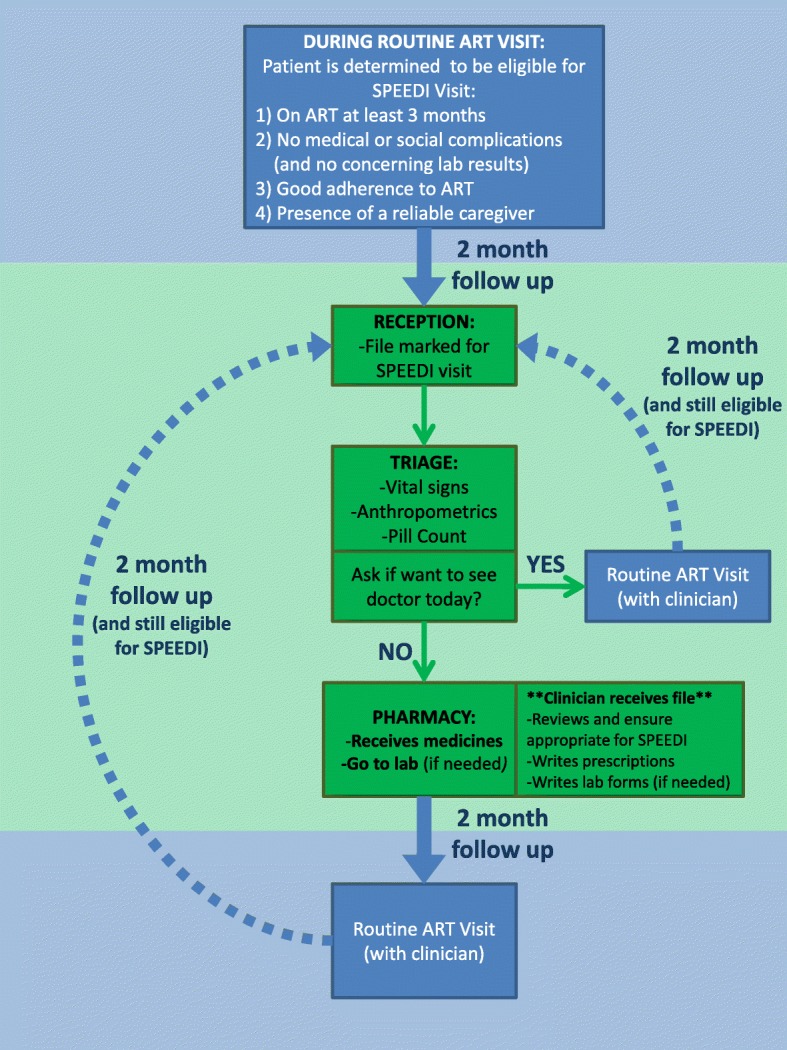
Standardized Pediatric Expedited Encounter for ART Drugs Initiative (SPEEDI) patient criteria and clinic flow during SPEEDI visits

SPEEDI targets clinically stable patients on ART by fast tracking and spacing out their ART visits. For a patient to qualify for a SPEEDI visit, the following criteria were met: 1) Age > 12 months and on ART for approximately 3 months; 2) no medical or social complications, and no concerning lab results; 3) good adherence to ART (defined as 95–105% adherence via pill counts, and via positive response during subjective questioning); and 4) presence of a reliable caregiver (Fig. 1).

During a SPEEDI visit, a file marked for SPEEDI during the previous visit is recognized at reception and placed in the designated SPEEDI bin. Triage team calls SPEEDI patients for vital signs, anthropometrics, pill counts, and ART adherence questioning as per normal clinic procedures. Patients with abnormal findings in triage are ineligible for a SPEEDI visit. While in triage, the patient and/or caregiver are asked if they wish to see a doctor, and if deferred proceed directly to pharmacy to collect medications. The patient file and triage data is reviewed by a clinician to ensure the patient is eligible for SPEEDI. If no issues are found, the clinician calculates medication dosages (and adjusts as needed for weight gain) and writes prescriptions for a 2 month supply. The file and prescriptions then proceed to pharmacy (where the SPEEDI patient/caregiver is waiting). Routine laboratory testing is done as needed during SPEEDI visits after the clinician reviews the SPEEDI file. SPEEDI patients are given two-month follow up visits, and alternate SPEEDI visits with routine visits, allowing them to be seen by a clinician at least every four months. Patients wishing to see a doctor, and those found not appropriate for SPEEDI visits, are seen by a clinician for a standard ART visit. While the majority of the COE patients are < 18 years old, there are no strict age restrictions for enrolment at the COE, and often adolescents and young adults > 18 years old received care at the COE. Therefore, an upper age limit was not an exclusion criterion for SPEEDI program, and young adults can also benefit from the program.

### Statistical analysis

We conducted a retrospective chart review on all COE patients on ART with at least one SPEEDI visit between 1st January 2013 and 31st December 2015. Age and time on ART was extracted from the patient’s first SPEEDI visit, while WHO Treatment staging (T-stage) and CD4 results were extracted from the most recent documented SPEEDI visit. Routine viral load testing did not begin in Tanzania until 2016; therefore, viral load data were not available for SPEEI patients and viral suppression could not be analyzed. Immunological classification groups were taken from World Health Organization (WHO) standards based on CD4 results and age [16]. Age data underwent Shapiro-Wilks tests to assess for normal distribution. “Good Outcome” was defined as patients still active in care and those successfully transferred out to another ART clinic, with clients who are transferred out being censored from the analysis at the transfer date; “Poor Outcome” was defined as deaths and LTFU. Outcomes data were extracted from the patient’s most recent clinic visit through 31 December 2015. LTFU was defined as patients on ART who had not been seen at COE for care for a period of 4 months or more from their scheduled COE follow up date. Mortality rates were calculated by dividing total deaths by the total time in years in which patients were in care, and are expressed as patient deaths per 100 years.

While no SPEEDI-specific patient surveys or interview questions were created, used, or implemented during the study period, we were able to utilize findings from Baylor Tanzania’s Annual Patient Satisfaction Survey results to assess overall trends in the survey results before and after initiating the SPEEDI program. The Baylor Tanzania Monitoring and Evaluation (M&E) teams conducted the annual “Patient Satisfaction Surveys” as part of routine M&E activities over a two-week period each year between 2011 and 2014. The annual patient satisfaction surveys were conducted via face-to-face questionnaire interviews at the COE by trained M&E staff with patients/caregivers who agree to participate during their regularly scheduled visits. Data collected from these routine annual M&E surveys is compiled into an annual patient satisfaction report, which is shared internally and used for existing quality improvement activities within the program. To broadly look for trends in patient satisfaction over the study period, specific data from questions focusing on time spent waiting in the COE and overall satisfaction with COE services from these surveys were analyzed. We compared survey results pre-SPEEDI implementation (May 2012) to post-SPEEDI implementation (May 2014) to assess for overall trends. There were no SPEEDI specific questions in the survey; however the following three question from the survey were of most interest and reviewed: “How long did it take to get all the services at the clinic?,” “Did the time spent at the clinic seem appropriate to you?,” and “How would you rate the clinic services overall?”

Annual mortality rates, deaths per 100 patient years, and LTFU rates of patients on ART at the Mbeya COE were calculated as part of Baylor Tanzania’s routine quarterly reporting since quarter four of 2011. These data were extracted from M&E quarterly reports at the COE for the years prior to SPEEDI implementation (2011–2012) and after SPEEDI implementation (2013–2015), and were analyzed to assess overall trends in these data in the pre- and post-SPEEDI eras of clinic operation. Study data were analyzed using STATA 11.2 (STATA Corporation, College Station, Texas, USA).

## Results

A total of 1164 pediatric, adolescent, and young adult ART patients utilized SPEEDI between January 2013 and December 2015, totaling 3493 SPEEDI visits. SPEEDI reached 51.3% (1164/2269) of the pediatric, adolescent, and young adult ART patients at the COE, and accounted for 7.7% (3493/44,489) of patient encounters during that time. The majority of SPEEI patients were female, older than 10 years of age, on ART for median time of 32 months, and predominately immunocompetent (“none” or “mild” by WHO categorization) (Table 1).

**Table 1:** Baseline Demographics, WHO treatment stage (T stage), and WHO immunologic classification of Standardized Pediatric Expedited Encounters for ART Drugs Initiative (SPEEDI) patients (*n* = 1164)

Characteristic	SPEEDI Patients (*n* = 1164)
% Females (n)	52.0% (605/1164)
Mean age (range)	11.8 years (1.2–25.5)
Age categories	
< 5yo	142 (12.2%)
5-9yo	322 (27.7%)
10-14yo	353 (30.3%)
15-18yo	251 (21.6%)
> 19yo	96 (8.2%)
Mean time on ART (range)	32 months (4–130 months)
WHO T-stages (%, n)	T1: 95% (1106/1164) N/A*: 5.0% (58/1164)
WHO Immunological classification of HIV-associated immunodeficiency [16] (%, n)	None: 68.7% (793/1155) Mild: 14.8% (171/1155) Advanced: 10.0% (115/1155) Severe: 6.6% (76/1155)

*“N/A” was assigned as a T-stage for patients on ART < 6 months. Those on ART for ≥6 months were assigned a T-stage

Regarding outcomes, 98.3% (1144/1164) of SPEEDI patients had good outcomes, with only 1.1% (14/1164) overall mortality and 0.6% (7/1164) LTFU (Table 2). The mortality rate of the SPEEDI patients were 0.61 deaths per 100 patient-years.

**Table 2:** Outcomes, loss to follow-up, and mortality rates of Standardized Pediatric Expedited Encounters for ART Drugs Initiative (SPEEDI) patients (*n* = 1164)

Indicator	SPEEDI Patients (*n* = 1164)
% Good outcomes (n) -Active in care (n) -Transferred out (n)	98.3% (1144/1164) −91.3% (1063/1164) −7.0% (81/1164)
% Poor outcomes (n) -Died (n) -Lost-to-follow up (n)	1.2% (14/1164) −1.1% (13/1164) −0.6% (7/1164)
Mortality rate	0.61 deaths per 100 patient-years

**Fig. 2:**
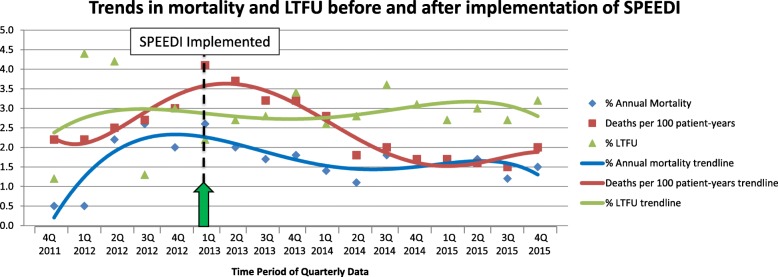
Trends in mortality and LTFU before and after implementation of SPEEDI

Overall trends in responses to the COE’s annual patient satisfaction survey comparing survey results from before SPEEDI was implemented (May 2012) to survey results after SPEEDI implementation (May 2014) showed improvements in patient reported wait times and overall satisfaction in clinic services. Specifically, percentages of patients reporting that they waited “greater than 1 hour” required to receive all services at the clinic fell from 63.1% in 2012 to 55.0% in 2014, percentage reporting their time spent at the clinic was “long” or “too long” fell from 35.8% in 2012 to 21.1% in 2014, and the percentage of patients rating overall services “good” or “excellent” remained high at 89.4% in 2012 to 90.5% in 2014. The annual mortality rate, deaths per 100 patient-years, and LTFU rate fluctuated greatly during the 2011–2015 time period, but overall trends of these three indicators showed stability or a decrease after SPEEDI implementation, most notably mortality and deaths per 100 patient-years (Fig. 2).

## Discussion

Our findings show that expediting clinic visits and spacing physician visits may be a safe and feasible model for ART delivery among stable pediatric, adolescent, and young adult ART patients in a resource limited setting. Using expedited visits in which patients on ART did not physically see a clinician led to high rates of good outcomes, low mortality, and low LTFU. Overall trends in the general patient satisfaction surveys results before and after initiating the SPEEDI program were encouraging, showing downward trends in reported clinic wait time and overall high satisfaction with clinic services during periods SPEEDI was implemented. We also noticed a general down trend in overall mortality of patients on ART at our clinic in the time period following implementation of SPEEDI. It was reassuring that our model of extending visit intervals and expediting ART visits in children overall produced good clinical outcomes, which are in line with findings in adult studies using expediting visits and appointment spacing for ART clinic visits to achieve good outcomes [7, 8, 17]. Our findings add important new information to the evidence base for differentiated models of care in children and adolescents – which currently is limited to and focusing on adherence clubs and community level models [18–24] – by specifically adding evidence on the facility-based, fast-track models which can compliment other pediatric differentiated care models.

Fast tracking pediatric and adolescents patients through SPEEDI has the potential to save time for both patients and clinicians. Such time saved allows clinicians to more efficiently use their time and skills for clinical duties such as caring for sicker, more complicated patients, as well as non-clinical duties such as reporting, continuous medical education, and quality improvement. By refocusing resources to patients most in need, there is potential for better allocation of resources, better patient care delivery, and reduced clinician burnout. Similar, the SPEEDI model has the potential to decongest clinics and make way for additional patients.

Economic concerns of patients (particularly transport costs) are a well known barrier to accessing care and a driver of poor retention to care and adherence to ART [25–29]. While not directly measured in our study, service delivery models that reduce the number and length of clinic visits benefits families by reducing costs associated with transportation and missed time from work [30, 31]. These costs, in addition to long wait times during clinic visits, are known systems-level barriers to accessing and adhering to HIV care in low and middle income countries [13], and resultant patient dissatisfaction can negatively impact retention to care. While our study only looked general patient satisfaction survey trends at our clinic – and not specific satisfaction with SPEEDI program – the broad results and trends were reassuring. The specific effects, including patient acceptance and satisfaction of the SPEEI program need more thorough investigation in the future.

Multi-month prescribing and expedited models of ART delivery for children and adolescents – such as SPEEDI – also have the potential to improve retention to HIV care. In our SPEEDI cohort, it was encouraging to see the low LTFU rates among SPEEDI patients (0.7%), which were lower than our COE’s average LTFU rate for all patients on ART (3.2%) as well as lower than published pediatric LTFU rates low and middle income countries [32]. Recent reports in Tanzania demonstrate LTFU rates in HIV positive children as high as 11–31% [33, 34], demonstrating the severity of the problem in our setting. The SPEEDI program, used in conjunction with high-quality comprehensive, multi-disciplinary, one-stop-shop services, and patient-first, child/adolescent-friendly attitudes – as is done at the Baylor COEs – provides an adaptable model of care that can be scaled out to help reduce these LTFU rates and improve clinical outcomes in children, adolescents, and young adults living with HIV.

**Fig. 3:**
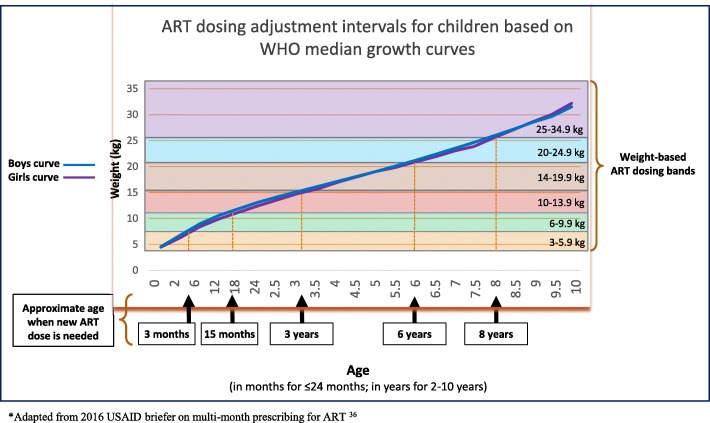
WHO Weight-for-age median growth curves and weight-based ART dosing bands for children demonstrating time intervals between ART dose adjustments in children*

Two major challenges to implementing expedited visits and appointment spacing for children on ART include limited or unpredictable ART drug supplies (particularly pediatric formulations) and concerns over growth monitoring and rapid weight gain in children necessitating dosing adjustments. Careful planning, forecasting, and good communication with national supply chain teams is crucial for any program/clinic implementing these models of care to ensure ART stock out does not occur. During implementation of SPEEDI, no treatment interruptions due to stock out occurred in SPEEDI patients due to the diligence of our pharmacy teams. SPEEDI also mandates that a child be weighed at every visit, so that weight and growth is documented every two months and dosing adjustments can be made. Interestingly, when comparing ART weight-based dosing bands to WHO growth curves for children [35], a child following the normal growth curve would only need an ART dose adjustment at around 3 months, 15 months, 3 years, 6 years, and 8 years of age; which are quite long intervals of time when only spacing ART appointments by 2–3 months (Fig. 3). As SPEEDI was piloted using two month refill visits as standard of care, additional value and potential at the facility and policy levels could be gain by adapting it to three month refill visits to reduce annual visits from six to four for stable patients.

This study has some limitations. The SPEEDI model selects for stable patients with good adherence. Patients who are clinically unstable, have significant co-morbidities, or have poor adherence have needs beyond what SPEEDI offers and, therefore, received more intensive services and were not included in this study. Moreover, the Baylor COEs are highly functioning, well-staffed pediatric clinics with a robust electronic medical records and M&E system, which may not reflect the reality of HIV clinics across sub-Saharan Africa. However, the SPEEDI model is designed so that it can be adapted and modified to fit the needs, abilities, and resources of any HIV clinic. While initial implementation of any novel clinic flow system requires more upfront time and effort, the downstream benefits of reducing clinic congestion and optimizing scarce clinician time is appealing to many over-burdened, under-resourced settings. Furthermore, our COE has ancillary services including social workers, counselors, and nutritionists that are available to augment patient cares. These services, which likely influenced patient outcomes in our setting, may not be available at other health facilities. Still, the general principles of our model of expedited and spaced out ART visits for children can be used at varying settings and adapted based on local contexts, needs, and resources.

Lack of viral load data is another important limitation of this study. We were unable to evaluate viral suppression as an outcome because viral load monitoring was not available to patients as Tanzania only recently rolled out routine viral load monitoring in 2016. Thus, good outcomes were approximated in SPEEDI patients using clinical status (healthy, thriving, no opportunistic infections, no clinical deterioration) for those who remained in care or who successfully transferred out. Therefore, an important next step to evaluate this program’s impact on achieving the third 90–90-90 target will be analyzing viral loads in SPEEDI patients. Early results from the SEARCH study in Uganda and Kenya are promising in demonstrating that streamlining ART clinic visits and spacing appointments for children can have high rates of viral suppression [36]. The direct effect of SPEEDI visits on patient satisfaction, mortality and LTFU was not directly measured in our study, and instead we relied on the data of our routine indictors during the study period. Therefore, it is difficult to know the influence and impact that can be attributed to SPEEDI on the observed trends of these indicators. Lastly, our program utilized two month follow up visits meaning patients will be examined by a physician every four months. Whether the program can be effective and sustainable by extending to three month follow up visits (thus six monthly physician exams) as suggested by recent WHO guidelines [4] is yet to be evaluated.

Nevertheless, this study helps lay the foundation for developing innovative ART delivery programs for children and adolescents and provides reassurance that expedited visits and appointment spacing can be safe and effective for children and adolescents on ART. In addition to delivering good clinical outcomes, SPEEDI can potentially decrease burdens felt by patients and health care providers. With expedited visits, there is potential for patients and families to experience decreased wait times and time away from school and/or work, ultimately leading to greater patient/caregiver satisfaction and retention to care [37]. With stable patients being diverted from regular physician visit, physicians could allocate more time to caring for complex patients. Additional research is needed to determine if SPEEDI has any direct associations with viral loads, patient satisfaction, motivating patients to adhere to ART, and provider burnout.

## Conclusion

The SPEEDI program is a potential service delivery model to both expedite and space out ART visits safely and effectively for children, adolescents, and young adults on ART. In our setting, SPEEDI patients had good clinical outcomes, low mortality, and low LTFU rates. Further research should target feasibility of implementation of such models on a larger scale in high-burdened, lower resourced settings, as well as integrating expedited visits and appointment spacing with other differentiated models, such as task shifting/sharing. As the global community moves towards universal ART coverage, SPEEDI can be an adaptable ART delivery model for children and adolescents living in a resource limited settings.

## Abbreviations

AIDS: Acquired Immunodeficiency Syndrome
ART: Antiretroviral Therapy
COE: Centre of Excellence
HIV: Human Immunodeficiency Virus
LTFU: Lost to follow up
M&E: Monitoring and Evaluation
NIMR: National Institute for Medical Research
SPEEDI: Standardized Pediatric Expedited Encounters for ART Drugs Initiative
WHO: World Health Organization
